# Understanding unplanned readmissions for children undergoing surgery in a single pediatric general surgical department

**DOI:** 10.1186/s12887-019-1672-7

**Published:** 2019-09-26

**Authors:** Chao Zheng, Hong Zhou, Hai Zhu, Bailin Chen, Lin Qiu, Chunbao Guo

**Affiliations:** 1 Department of Pediatric General Surgery, Children’s Hospital, Chongqing Medical University, Chongqing, People’s Republic of China; 2 Ministry of Education Key Laboratory of Child Development and Disorders, Children’s Hospital, Chongqing Medical University, 136, 2nd Rd. Chongqing, Zhongshan, 400014 People’s Republic of China

**Keywords:** Unplanned readmissions, General surgical specialties, Hospitalization

## Abstract

**Background:**

The aim of the current research was to investigate the unplanned readmission rates and identify the risk factors of unplanned readmissions in pediatric general surgical specialties.

**Methods:**

A retrospective review of unplanned readmissions following initial surgery from July 1, 2010, to June 30, 2017, in the general surgical specialties at an academic tertiary care hospital was performed. The main outcome of interest was unplanned readmission rates, the common causes for readmission. The risk factors involved in the unplanned readmissions were further investigated using univariate and multivariate analyses.

**Results:**

Of the 3263 patients who underwent surgery and discharge, 176 (9%) were unplanned readmissions. The most frequent surgical operation related to readmission was appendectomy, and the common readmission causes were associated with treatment of gastrointestinal complaints/complications. Multivariable analysis demonstrated that emergency surgery (*p* = 0.016, odds ratio [OR] = 2.73; 95% CI = 1.35–6.19), major complications (*p* = 0.042, OR = 2.43; 95% CI = 1.12–4.71) and the initial hospital length of stay (*p* = 0.036, OR = 3.46; 95% CI = 1.67–7.53) were independent risk factors for readmission.

**Conclusions:**

This study identified potential risks for readmission, which should be targeted for interventions to improve quality and resource allocation.

## Background

Recently, hospital readmission, the important metric for the quality measurement of patient care, was pushed forward due to pressure linked to payment [1–3]. Furthermore, a patient’s unplanned readmission influences their quality of life and limits hospital resources. In 2008, the Medicare Payment Advisory Commission in the USA conducted payment reforms to encourage a reduction in unnecessary surgical readmissions and save health care costs [4, 5].

For surgical patients, the postoperative complications were the most reasons related to hospital readmission, which is fundamentally different from medical patients [6]. Because specific procedures carry high readmission risk, the causes of readmission for surgical patients are often associated with underlying surgical conditions, suggesting that there is an opportunity to identify the patients who carry the risk of readmission postoperatively and intervene this risk preoperatively.

Many studies have been carried out to investigate the risk profiles of readmission within precise patient populations, including older age patients [7–9]. These previous studies highlight that the readmissions might be preventable with comprehensive preoperative and postoperative care. However, there was a few research that point to unplanned surgical readmission among pediatric patients, and it was unclear whether all the reported factors carry the similar risk for surgical readmission among pediatric population.

The purpose of the current research is to examine the readmitted pediatric patients undergoing general surgical operations and define the factors related to unplanned readmission in the general surgical department among pediatric patient population.

## Methods

### Patients and methods

The current research is performed utilizing a patient database in the general surgical department at Chongqing Medical University Children’s Hospital from July 1, 2010 to June 30, 2017. The electronic medical records of all patients for any unexpected readmissions within 30 days of the inpatient general surgery procedures (Table 1), and any documented reasons for the readmission were reviewed with two independent investigators who underwent our training. All the case and control samples and evidence of complicated appendicitis. The patients should age more than one month and less than 16 years. The unplanned readmissions were defined as readmissions for hospitalization occurring within a 30-day period that starts on the day the patient is discharged, which is as a result of a complication directly or indirectly related to the initial surgical procedure regardless of whether it occurred within the same hospital admission. We excluded patients if two-stage surgeries or outpatient procedures were planned and if the readmission was the necessary planned treatment following their initial operation (like, chemotherapy). The patients death within 30 days of initial operation were also excluded for the readmission risk analysis.

**Table 1:** Initial surgical compositions of unplanned readmission and its matched controls

Type of initial surgery	Unplanned readmission	Control	Total cases (%)
Kasai procedure	35	66	887 (3.9)
Hernia corrective surgery	8	14	616 (1.3)
Cholecystectomy	5	10	213 (2.3)
External drainage of biliary dilatation	9	17	147 (6.1)
Hepatic surgery	2	4	28 (7.1)
Roux-Y anastomosis	8	13	374 (2.1)
Appendectomy	72	139	413 (17.4)
Meckel diverticulum	5	11	194 (2.6)
Devascularization for CTPV	4	8	41 (9.8)
Trauma laparotomy	2	2	25 (8.0)
Laparotomy	21	42	250 (8.4)
Pancreatic operation	4	10	48 (8.3)
Fistula close procedure	1	2	27 (3.7)
Total	176	338	3263 (5.4)

*CTPV* Cavernous transformation of the portal vein

During the same period at the same institution, the patients as control were identified from patients underwent the same operation without unplanned readmission as a given case with unplanned readmissions. In the corresponding case, we randomly selected two immediately preceding patients who underwent the same principal procedure within six-month period around the surgery date. After reviewing the entire medical record, the independent investigators determined the reasons for the readmission according to the standard International Classification of Diseases (ICD-9), Ninth Revision code (e.g., surgical site infection [SSI], postoperative ileus).

### Study covariates

We primarily evaluated the rate of unplanned readmission across the various specialties. We further investigated the factors related to unplanned readmission. We collected over 20 explanatory perioperative characteristic variables to explore the reasons for readmission, including patient demographics, preoperative morbidity variables, intraoperative variables, postoperative characteristics, including 30-day postoperative complications and unplanned readmission in the inpatient setting. The postoperative complications, which should be related with readmission, were further determined.

### Statistical analysis

The data manipulations were performed using SPSS Version 20 (IBM Corp, Armonk, NY). First, we generated descriptive statistics to explore the frequency of unplanned readmissions following the initial general surgical procedures. Univariate analysis was utilized to examine patient explanatory perioperative variables among the patients with and without unplanned readmission. Continuous data with normal distribution were presented as the mean ± (standard deviation) and assessed with a Student t test, and nonnormally distributed variables were presented as the median (interquartile ranges) and tested using the Mann-Whitney U test. Categorical data were expressed as frequencies (percentages) and analyzed using a Fisher’s exact test or χ2 test, as appropriate. To determine independent factors related to unplanned readmission, Multivariable analysis was further carried out. Variables associated with unplanned readmissions with *p* value ≤0.20 in univariate analyses were forced into the multivariable analysis. Statistical significance was recognized if 2-sided *P* values were less 0.05.

## Results

### Patient readmission features

There were 225 patients with readmission among 3263 patients who underwent general surgery procedures in our institute, with an overall readmission rate of 6.9%, and 49 (1.5%) readmissions were excluded because they were planned (18), unrelated (21) or self-discharged (interrupted managements due to some socioeconomic reasons) (10) at the index admission. Table 1 summarizes the features of readmissions, including the rates of readmission for specific diagnoses. There were 176 (5.4%) unplanned readmissions, with an average age of 3.6 years.

### Procedures

Unplanned readmission occurred following 22 different types of initial operations. Among them, 2 procedures accounted for almost half of all the unplanned readmissions. Readmission frequencies stratified by procedure type within our cohort are detailed in Table 1. The readmission rates for individual procedures ranged from 1.3% (8/616) after hernia corrective surgery to 17.4% (72/413) after appendectomy.

### Reasons for unplanned readmission

To evaluate for potentially preventable readmission, we then focused on reasons for unplanned readmission in pediatric general surgery patients. The common reasons for unplanned readmission were related to treatment for gastrointestinal (GI) complaints and complications (intestinal obstruction, nausea, vomiting, etc.) (31.8%, 56/176), postoperative biliary tract infection (18.2%, 32/176) and surgical infections, including ileus (14.2%, 25/176) or intra-abdominal abscess and infection (13.6%, 24/176), followed by postoperative electrolyte disturbance (3.4%, 6/176), and hernia relapse (8.5%, 15/176). Among these reasons, gastrointestinal (GI) complaints and complications combined with postoperative biliary tract infection accounted for approximately half of all readmissions (Table 2).

**Table 2:** Reasons for unplanned readmission

Indication for readmissions	First readmission	Median date of readmission (range)
Alimentary tract hemorrhage	7	7 (1,29)
Anastomotic leakage	4	4 (3,6)
Wound dehiscence	6	5 (2,7)
Surgical site infection	19	15 (7,26)
Intra-abdominal abscess and infection	14	9 (5,29)
Postoperative electrolyte disturbance	6	6 (1,15)
Postoperative biliary tract infection	21	18 (6,30)
Postoperative ileus	16	15 (5, 27)
Heinia relapse	15	23 (18,28)
Other	7	21 (3,25)
Total	176	36 (1, 30)

The 18th day (1–30 days) after surgery was the median date for unplanned readmission (Table 2). Five of 176 cases (2.8%) were readmitted the first day after surgery. The interquartile range of unplanned readmission was 16 days for GI complaints/complications, 21 days for postoperative biliary tract infection, and 14 days for intra-abdominal abscess and infection. When examining early unplanned readmission (within 7 days of discharge), a total of 25 (4.0%) patients were readmitted. The top three reasons for early unplanned readmission were different (postoperative electrolyte disturbance, anastomotic leakage, and bleeding). Prior to readmission, the majority of patients with early unplanned readmission had an consultation with the initial surgical team.

### Case-control analysis for risk factors of readmission

Under univariate analysis, factors related to unplanned 30-day readmission included operative blood loss (*p* = 0.039), surgeon’s skill level (*p* = 0.048), emergency surgery (*p* = 0.004), major complications (*p* = 0.008), infectious complications (*p* = 0.025), contaminated or dirty wound (*p* = 0.047) and the initial length of hospital stay (*p* = 0.011) (Table 3). No statistically significant differences were noted in terms of age, sex, and operative time.

**Table 3:** Univariate analysis of factors associated with unplanned readmission

	Total population		
	Control (338)	Unplanned readmission (176)	*p* values
Age (yrs)	3.58 ± 3.63	3.56 ± 3.73	0.96
Male: female	216:122	107:69	0.49
Operative time (mins)	96.9 ± 55.2	105.5 ± 58.6	0.10
Operative blood loss (mL)	9.3 ± 5.1	12.7 ± 8.3	0.039
Emergency surgery, n (%)	141 (41.7)	96 (54.5)	0.004
Initial surgery-related risk level, n (%)			
Low	206 (60.9)	95 (54.0)	0.18
Intermediate	107 (31.7)	70 (39.8)	
high	25 (7.4)	11 (6.3)	
ASA classification, n (%)			
ASA1	39 (11.5)	15 (8.5)	0.35
ASA2	171 (50.6)	90 (51.1)	
ASA3	123 (36.4)	66 (37.5)	
ASA4	5 (1.5)	5 (2.9)	
Surgeons level, n (%)			
Resident	85 (25.1)	47 (26.7)	0.048
Senior Resident	82 (24.3)	31 (17.6)	
Fellow	125 (37.0)	83 (47.2)	
Attending	46 (13.6)	15 (8.5)	
Operation on weekend, n (%)	53 (15.7)	30 (17.0)	0.69
Time of Case Start (6 pm-7 am), n (%)	124 (36.7)	57 (32.4)	0.33
Contaminated or dirty wound, n (%)	132 (39.1)	83 (47.2)	0.047
Major complications,n(%)	58 (17.2)	16 (9.1)	0.008
Infectious complications, n(%)	91 (26.9)	33 (18.8)	0.025
The initial hospital length of stay (days)	10.2 ± 5.7	12.9 ± 5.3	0.011

Multivariable independent risk factors of unplanned readmission are shown in Table 4. After adjusting for other characteristics, independent predictors of readmission were emergency surgery (*p* = 0.016, OR = 2.73; 95% CI = 1.35–6.19), major complications (*p* = 0.042, OR = 2.43; 95% CI = 1.12–4.71) and the initial length of hospital stay (*p* = 0.036, OR = 3.46; 95% CI = 1.67–7.53). The surgeon’s skill level, which was related to the unplanned readmission in univariate comparison (*p* = 0.048), was no longer significant association with the unplanned readmission in the multivariate analysis.

**Table 4:** Multivariable analyses of the factors with unplanned readmission

Scheduled	*p* values	OR	95% CI
Emergency surgery	0.016	2.73	1.35–6.19
Major complications	0.042	2.43	1.12–4.71
The initial hospital length of stay	0.036	3.46	1.67–7.53

The variables in the multivariable analyses included operative time, operative blood loss, emergency surgery, initial surgery-related risk level, surgeons level, contaminated or dirty wound, major complications, Infectious complications, the initial hospital length of stay. Only significant risk factors for related or unrelated readmissions are shown (*P* < .005)

## Discussion

With the increased attention placed upon readmission, it is essential to understand risk factors to deliver postoperative care for quality improvement, which has expanded to pediatric postoperative care [10–12]. The current study represents the first investigation to explore the contributing risk factors for unplanned readmission toward the development of strategies in pediatric patients who have undergone a variety of operations in a general surgical department. We demonstrated that the unplanned readmission were associated with more operative blood loss, faced a health emergency, or had more postoperative complications. Across pediatric surgical specialties, the common reasons for unplanned readmission were postoperative ileus and adhesion, postoperative biliary tract infection and wound problems.

Our overall readmission rate of 6.9% was consistent with the previously published readmission rate of 5.2% after general surgical procedures in pediatric population [13]. In the USA, for general pediatric surgery, the unplanned readmission rates are 4.4–6.5% [14, 15]. Differences in sample size and cases in the aforementioned studies compared with the current study may account for some of the differences in the readmission rates. You know, in China, due to the economic backward and imperfect of health insurance, readmissions may be influenced because some of patients could not afford the medical expense and select discharge in advance. The self-discharged patients implied the managements were interrupted, which should be the risk for the unplanned readmission. The Pediatric National Surgical Quality Improvement Program (NSQIP-P) includes major procedures that might bias toward higher reports of readmission for increased frequency of the occurrences of postoperative complications. In the current data, the appendectomies and laparotomy operations were the most common surgeries, which were related to unplanned readmission. A similar distribution of procedures, involved in unplanned readmission was present in a number of other studies [16, 17]. Reducing readmission rates and designing interventions would be important for pediatric surgical patients. The current 1.5% of planned readmission was consistent with that reported by others [18].

For quality improvement, identification the greater risks carried by which postoperative complications would be beneficial to prevent readmissions. Our research provided a retrospective unplanned readmission review and determined the reasons for readmission. While the reasons for unplanned readmission varied by different specialty, the current research supported the conclusion that postoperative ileus and adhesion were the most common reasons for readmission within general pediatric surgery, which was similar to the reported reasons of others [13, 14]. Gastrointestinal-related causes were the most common reasons for readmission, accounting for one-third of readmissions. Consequently, the most frequently performed procedure during readmissions was enterolysis. This finding contrasts with the findings for adults, for whom surgical site infections represented the leading cause of readmission among general surgery procedures [19]. In our institute, the adhesion events were treated with cautions. Some mild obstructive symptoms were readmitted for fluid resuscitation, which might account for the current high rate of post-operative intestinal adhesions. The indication for postoperative intestinal adhesions involved fluid resuscitation as well as attitudes toward readmission warrant further investigation and present opportunities for quality improvement. After postoperative intestinal adhesions, postoperative surgical site infection (SSI) was the next most frequent cause related to readmission. Recently, it was reported that over 50% of the patients following general surgical procedure readmitted due to a SSI [20]. Additionally, an operative time greater than 4 h conferred increased risk for SSI [20]. Operative approach and the transfusion of blood products depend on clinical manifestation and surgical judgment and technique. To decrease the readmissions, multidisciplinary efforts by operative surgeon, physicians and nurses should be focused on these modifiable risk factors perioperatively.

Our study provides further evidence that operative blood loss, emergency surgery and complications are the most predictive for hospital readmissions [2, 21]. Most risk factors, such as severe condition and massive surgery, were either nonmodifiable comorbidities or markers for severe underlying illness, which is not surprising. For instance, emergency surgery was a risk factor in our study [1]. However, in this research, less intuitive factors were also associated with substantial risk, such as longer length of stay and different surgeon skill levels. We found that readmission was two times when the procedure was performed with higher level surgeons. Although some authors have not found this association, which should be a potentially causative modifiable risk factor, this finding implied that strict quality control should be undertaken in our institute. After general surgery procedures, it was identified that shorter length of stay was predisposed toward readmission in some research [22]. In fact, the current data suggested that multiple complications was associated with longer length of stay and more readmission, which was also present following bariatric surgery and colorectal surgery [23, 24]. The patient’s comorbidities should drive the longer length of stay, which might also account for the readmission increase. Implications the current finding is that many of readmissions may be avoidable in a certain part of patients, which presents an opportunity to improve quality and the delivery of outpatient care or improved educational processes in our institute. The insight obtained from readmission can help support or refute the efficacy of a protocol applied across the initial operations and develop new surgical methods in the context of regular complication control. In addition, the lessons learned can be of great value in teaching young clinicians [25].

There are limitations in certain aspects of our study. First, our study has a limitation inherent to retrospective nature wherein selection bias could be confounding, which might restrict the generalizability of the current conclusions. The current data were gathered from our single general surgical department, reflecting the experience only from our institution. Although most of patients are back to our institution for further care, some of readmittion might get into local hospitals. Thus, the current real readmission may be higher than the estimated one. Finally, the lack of readmission information for specific operation is another weakness of the current research, which might help to identify high risk of operation for readmission.

## Conclusion

We uncovered the common reasons for unplanned readmissions at our institution, namely, infection and hemorrhage. We also identified the risk factors for unplanned readmissions following general surgery in a pediatric patient population. A standardized, well-defined registry of all unplanned readmissions classified by type of index operation is important for the care quality improvement across a large population of infants and children.

## Data Availability

The datasets during and/or analyzed during the current study are available from the corresponding author on reasonable request.

## Abbreviations

CI: Confidence interval
GI: Gastrointestinal
OR: Odds ratio
SSI: Surgical site infection
